# Gastrointestinal Cancers with Consideration of DPD and UGT1A1 Plasma Levels: Chemotherapy-Related Toxicity

**DOI:** 10.3390/life15071071

**Published:** 2025-07-04

**Authors:** Velko Minchev, Hristo Tsankov, Bozil Robev, Martin Takov, Stefan Federchev, Kalina Kamenova, Lozan Todorov, Liliya Atanasova, Nadya Hristova-Avakumova, Rumen Nikolov, Pavlina Gateva, Vanyo Mitev

**Affiliations:** 1 Department of Medical Oncology, SofiaMed University Hospital, 1113 Sofia, Bulgaria; 2 Hematology Clinic, Department of Medical Oncology, UMHAT “St. Ivan Rilski”, 9002 Sofia, Bulgaria; 3 Faculty of Pharmacy, Medical University—Sofia, 1431 Sofia, Bulgaria; 4 Medical Faculty, Medical University—Sofia, 1431 Sofia, Bulgaria; 5 Department of Medical Chemistry and Biochemistry, Medical Faculty, Medical University—Sofia, 1431 Sofia, Bulgaria; kkamenova@medfac.mu-sofia.bg (K.K.);; 6 Department of Chemistry, Faculty of Pharmacy, Medical University—Sofia, 1431 Sofia, Bulgaria; 7 Department of Medical Physics and Biophysics, Medical Faculty, Medical University—Sofia, 1431 Sofia, Bulgaria; 8 Department of Pharmacology and Toxicology, Medical Faculty, Medical University—Sofia, 1431 Sofia, Bulgaria; 9 Research Institute of Innovative Medical Science, Medical University—Sofia, 1431 Sofia, Bulgaria

**Keywords:** gastrointestinal malignancies, FOLFOX, FOLFIRI, dihydropyrimidine dehydrogenase, UDP-glucuronosyltransferase, myelotoxicity

## Abstract

Unpredictable, dose-limiting toxicity remains a challenge in cancer treatment. We evaluated dihydropyrimidine dehydrogenase (DPD) and UDP-glucuronosyltransferase 1A1 (UGT1A1) plasma levels in the context of chemotherapy-induced toxicity and disease progression. Seventy gastrointestinal cancer patients (30 FOLFOX; 40 FOLFIRI) were enrolled. DPD and UGT1A1 plasma levels were determined using ELISA. Univariable and bivariable analyses and a general linear model (GLM) framework were used. Post-infusional reductions in white blood cell and granulocyte counts were observed. For FOLFOX, the granulocyte counts decreased by 17% (r = 0.54; *p* = 0.0030), while FOLFIRI caused a 41% reduction (r = 0.43; *p* = 0.0063). DPD levels were lower in FOLFOX than in FOLFIRI (2.543 vs. 3.579; *p* = 0.0363; Cohen’s d = 0.52). The multiple linear regression models associated DPD levels with cancer progression (b* = 0.258, *p* = 0.034). The bivariate analysis and multiple linear regression indicated some trends of association between UGT1A1 levels and reduction in white blood cell (b* = 0.359, *p* = 0.042) and granulocyte counts (b* = 0.383, *p* = 0.030) among FOLFIRI-treated patients. These preliminary observations suggest that DPD and UGT1A1 might contribute to evaluating response assessment.

## 1. Introduction

Gastrointestinal (GI) cancers refer to various types of malignancies affecting different parts of the digestive system from the esophagus to the stomach, the pancreas, the liver, the small intestine, the colon, the rectum, and the anus. They represent a major global public health concern, accounting for a significant portion of cancer-related morbidity (26% of all global cancer cases) and mortality (35% of cancer-related deaths) [1]. Along with the concern regarding the increased incidence of GI malignancies over the last few decades, another factor that has drawn significant attention is the early age of onset [2]. This increasing number of newly diagnosed cases occurring in younger populations was found predominantly for gastric, pancreatic, and colorectal malignancies [3]. The occurrence of gastrointestinal (GI) cancers is strongly correlated with a range of risk factors. While some factors are unique to specific cancer types, others, such as age, lifestyle habits, and genetic predisposition, are common for all forms of these malignancies [4,5].

Treating GI cancer is a complex process that requires a multidisciplinary strategy and involves multiple healthcare professionals. Clinicians individualize the therapeutic approach according to the specific cancer type, its stage, and the patient’s overall health status. Despite the significant advances in developing novel anticancer agents targeting specific molecular pathways and genetic mechanisms, conventional chemotherapy remains the most widely employed cornerstone of therapeutic intervention [6]. Patients’ response to chemotherapy can be highly variable due to individual variations in drug metabolism and detoxification pathways. The priorities of research are to explore strategies for developing rapid, cost-effective, and non-invasive blood tests that detect markers indicating drug metabolism efficiency and potential toxicity. In particular, the enzymes dihydropyrimidine dehydrogenase (DPD) and UDP-glucuronosyltransferase 1A1 (UGT1A1) under investigation in this work are essential for the metabolic breakdown of fluorouracil (5-FU) and irinotecan. Both drugs remain standard treatments for somatic tumors, and variations in these enzymes have been linked to therapy effectiveness and myelotoxicity (also studied here).

Both irinotecan and fluorouracil (5-FU) have proven effective as monotherapies or in different combinations in treating solid tumors, including GI malignancies. The FOLFOX regimen (folinic acid, fluorouracil and oxaliplatin) and the FOLFIRI protocol (folinic acid, fluorouracil and irinotecan) are widely recognized for their efficacy as standard chemotherapy regimens. 5-FU is a heterocyclic aromatic organic compound, being a pyrimidine analog composed of a pyrimidine and a furan ring. It blocks the enzyme thymidylate synthase, resulting in an intracellular thymine deficit, the inhibition of DNA synthesis, and the induction of cytotoxic effects. After 5-FU penetrates the cell, it undergoes two competitive processes: catabolic inactivation and anabolic generation of active metabolites. The catabolic pathways in the liver account for the metabolism of 80% to 85% of the injected 5-FU. Within the anabolic pathway, only 1 to 5% of the injected 5-FU is transformed into cytotoxic substances [7,8].

Irinotecan is a bioactive compound and an analog of camptothecin. It is an alkaloid derived from *Camptotheca acuminata* (a tree native to China) belonging to a class of chemotherapeutic agents known as topoisomerase inhibitors. These agents interact with topoisomerase I and II (enzymes that control changes in DNA structure, replication, and transcription). Topoisomerase-I generates single-strand breaks in DNA, preventing supercoiling. Irinotecan is metabolized in the hepatic pathway by carboxylesterase to the drug metabolite SN-38. The latter is a much more potent topoisomerase-I inhibitor, determining efficacy and toxicity [9,10].

The main challenges in the treatment of cancer are associated with the limitations of the medications currently in use, including a small therapeutic range, dose-limiting toxicity, and the development of resistance. Toxic effects after 5-FU administration are most pronounced in the cells of the gastrointestinal mucosa (toxic diarrhea in 75% of patients), the bone marrow (neutropenia, leukopenia, thrombocytopenia in 69% of patients), and, less frequently, cause cardiac complications [11,12,13]. Irinotecan side effects vary: severe diarrhea occurred in 26% of patients on monotherapy, but only in 11% on the FOLFIRI regimen. In contrast, the influence of neutropenia of any grade was similar in the two groups: 63% in the monotherapy cohort and 61% in the FOLFIRI group [14]. When used as monotherapy, response rates for 5-FU and irinotecan range from 10 to 30% and 18 to 32%, respectively, but improve significantly when combined or used with other agents [15,16,17]. The complexity of managing overlapping toxicities from the combined use of both drugs underscores the need for predictive markers to improve treatment personalization.

An ultimate goal in modern cancer therapy is to find the balance of “effectiveness–safety–price” combined with a personal approach. Strategies include maximizing multimodal treatment and diagnostic procedures in clinical practice. Among the key challenges in cancer therapy, drug resistance remains a leading cause of treatment failure. When evaluating the expected benefits related to treatment effectiveness and survival rate, attention is drawn to the issue of the safety of the applied therapy and the frequency of side and toxic effects. The leading strategy is to study their prediction, control, and prevention potential [18,19].

There is a 30% inter-individual variation in irinotecan clearance (CL), whereas SN-38 clearance shows a significantly more considerable variation of 80% [20]. For 5-FU, the inter-individual variability among cancer patients was 27%, with a mean CL value of 235 l/h [21]. Several factors could be involved in the pharmacokinetic variability of drugs. The three main cited factors are environmental factors (other medications, lifestyle), physiological factors (elderly patients, obesity, liver and kidney function), and pharmacogenetic factors. Hereditary polymorphisms have been shown to influence pharmacokinetics, pharmacodynamics, and individual toxicity associated with antitumor therapy [22]. There are over 30 polymorphisms associated with the deficiency of dihydropyrimidine dehydrogenase, an important enzyme related to the metabolism and toxic effects of 5-FU treatment. Two missense polymorphisms in GSTP1 cause reduced glutathione-S-transferase enzyme activity and neuropathy associated with oxaliplatin treatment. Dose-dependent neutropenia is often reported in patients homozygous for the seven-fold repeat allele of UDP-glucoronosyl-transferase (UGT1A1*28) [23,24].

From this point of view, assessing adverse effects associated with 5-FU and irinotecan administration and exploring their relationship with the level of plasma enzymes, key indicators for chemotherapy-related toxicity and drug metabolism, is important in terms of interindividual variability in pharmacokinetics and drug-induced toxicities. Therefore, this study aims to evaluate dihydropyrimidine dehydrogenase (DPD) and UDP-glucuronosyltransferase 1A1 (UGT1A1) plasma levels in the context of chemotherapy-induced toxicity and estimate their relationships with hematological side effects and disease-specific variables.

## 2. Materials and Methods

### 2.1. Patients and Clinical Information

#### 2.1.1. Study Design

This study was conducted at the “Medical Oncology” department of SofiaMed University Hospital, Sofia, Bulgaria. Patients with histologically confirmed gastrointestinal malignancies were included. The study focused on cancer patients approved for FOLFOX or FOLFIRI chemotherapy regimens. The FOLFOX and FOLFIRI regimens follow a standard protocol of folinic acid (400 mg/m^2^) and fluorouracil (400 mg/m^2^ bolus + 2400 mg/m^2^ infusion over 46 h). The main difference is in the additional cytotoxic agent, which is oxaliplatin (85 mg/m^2^) for FOLFOX and irinotecan (180 mg/m^2^) for FOLFIRI. Both regimens follow a treatment schedule with a 2-week interval between each cycle according to the guidelines of BOND from 2018 (Bulgarian oncology scientific Society) and ESMO from 2023 (European Society for Medical Oncology) [25,26]. On the days of pre- and post-treatment hematological assessment (defined by the BOND guidelines), supplementary blood samples were collected together with routine blood work for experimental use. On the day of the first chemotherapeutic infusion, a test blood sample was collected from each patient prior to the intravenous therapy to assess their baseline condition and confirm their eligibility to begin the therapy protocol. The sample collected on the day of the first infusion was also used to determine the DPD and UGT1A1 enzyme levels. Prior to the second infusion, physicians ordered additional hematological and biochemistry tests to monitor the patient’s condition. The follow-up blood samples were collected on the day of the second infusion, 14 days after the first one. The parameters from this follow-up test were analyzed and correlated with the baseline levels and the level of enzymes of crucial importance to metabolize the chemotherapy drugs 5-FU and irinotecan—DPD and UGT1A1. The recruitment period for patients on the FOLFOX regimen was 12 months (from September 2019 to September 2020), while for those treated with FOLFIRI, the recruitment period was 18 months (from March 2022 to September 2023). The research received approval from the Institutional Ethical Review Board of Medical University–Sofia, and it was performed according to the Declaration of Helsinki.

#### 2.1.2. Patient Recruiting Procedure

The eligibility criteria for participation in the study were as follows: being aged over 18, having solid GI tumors with histological verification from a pathologist, and being scheduled for chemotherapy with FOLFOX or FOLFIRI. Exclusion criteria included there being insufficient patient data for the study’s objectives and information regarding another malignancy in the patient’s medical records. Information concerning symptoms, comorbidities, family history, lifestyle habits, and allergies was collected from patients. Examinations included general condition assessment, BMI estimation, blood pressure and pulse measurements, and ECG. Evaluation of patients’ activity level in relation to their disease, i.e., ECOG (Eastern Cooperative Oncology Groups) performance status, was performed. Laboratory testing, imaging studies, and histological examination with TNM staging were conducted as part of the baseline assessment. The participants were informed about the study. Written informed consent was obtained from each patient.

#### 2.1.3. Key Parameters of the Studied Groups of Patients

The basic features of the analyzed patient cohort were gender, age, disease characteristics associated with initial tumor localization, histological subtype and histological grade, the presence or absence of regional or distant metastases (N and M status), ECOG performance status, and laboratory test values (white blood cell count, lymphocytes count, granulocyte count, platelets, red blood cell count, hematocrit, hemoglobin, creatinine, total bilirubin, aspartate aminotransferase, and alanine aminotransferase). Given the fact that all patients on the FOLFIRI regimen were in stage IV, whereas patients treated with FOLFOX included those in earlier stages, the platelet-to-lymphocyte ratio (PLR) and the lymphocyte-to-monocyte ratio (LMR) were estimated. Both have been frequently cited in the literature as markers of systemic inflammation and hematological alterations influenced by disease stage and have prognostic significance in various malignancies, including GIT cancer [27,28].

### 2.2. Dihydropyrimidine Dehydrogenase (DPD) and UDP-Glucuronosyltransferase (UGT1A1) Enzyme Level Determination

An enzyme-linked immunosorbent assay was used to assess blood plasma levels of dihydropyrimidine dehydrogenase (DPD) in patients treated with the FOLFOX or the FOLFIRI regimen. UDP-glucuronosyltransferase 1A1 (UGT1A1) levels were measured only in patients undergoing FOLFIRI treatment. UGT1A1 testing was not necessary for the FOLFOX regimen since it does not include irinotecan (the drug primarily associated with UGT1A1-related toxicity).

The Dihydropyrimidine Dehydrogenase ELISA Kit (Catalog No. EH2958; Wuhan Fine Biotech Co., Ltd. (FineTest), Wuhan, Hubei, China; range: 0.156–10 ng/mL; sensitivity: 0.094 ng/mL), procured in 2019 and 2023, was used to measure patients’ plasma DPD levels. For the determination of the human UGT1A1 enzyme level, the UDP-glucuronosyltransferase 1-1 ELISA Kit (Catalog No. EH1469; Wuhan Fine Biotech Co., Ltd. (FineTest), Wuhan, Hubei, China; range: 0.156–10 ng/mL; sensitivity: 0.094 ng/mL) was used. For each ELISA Kit, standard curves were constructed using manufacturer-supplied standards with known concentrations. Absorbance readings for the standards and patient samples were taken at 450 nm using BioTek ELx800 Absorbance Microplate Reader (BioTek, Winooski, VT, USA). Patients’ enzyme levels were calculated using the respective standard curve equations.

### 2.3. Statistical Analysis

Patients’ data were collected and organized into a structured database using Excel. Several mathematical and statistical methods were employed when analyzing the obtained experimental data. Quantitative variables were evaluated using variation analysis. Key statistical parameters, i.e., the mean value, median, range, and standard deviation, were determined in order to obtain information concerning data central tendency, and dispersion. Frequency analysis of qualitative variables was performed by estimating the absolute and relative frequencies. Statistical hypothesis testing was performed using the paired Wilcoxon test, Student’s unpaired *t*-test, and the chi-square (χ^2^) test. The statistical significance of the differences between the means for the compared dependent or independent groups was determined, and the examination of associations between categorical variables was performed. A critical significance level was set at 0.05 (ns—not significant; * *p* < 0.05; ** *p* < 0.01; *** *p* < 0.001; **** *p* < 0.0001). Data processing and statistical analysis were performed using Microsoft Excel, G*Power 3.1.9.7, and GraphPad Prism 6. The effect size and Cohen’s d were estimated to quantify standardized mean differences. Cohen’s d values were interpreted as follows: 0.2: small effects: 0.5: a medium effect; and 0.8: a greater or large effect [29]. IBM SPSS statistics version 26.0 (IBM Corp., Armonk, NY, USA) was used to conduct analyses within the general linear model framework. The employed modeling strategy was consistent with that of Garg et al., 2012 [12].

## 3. Results

After collecting informed consent and comprehensive information covering all key parameters of the studied patient group, we analyzed the clinical and biochemical data and the DPD and UGT1A1 levels obtained from the ELISA experiments performed as described in the Materials and Methods. Following data organization, we applied the statistical tests outlined in Section 2 and examined differences, correlations, and significance trends. The clinical and experimental data and their interpretations are presented in detail in this section.

The study comprised 70 patients: 38 males (54.29%) and 32 females (45.71%). The mean age was 64 years and 9 months (range from 34 to 83). Most patients were older adults, with a significant proportion of cases in individuals in the age range groups of 60 to 69 (30%, 21 patients) and 70 to 79 (35.71%, 25 patients). Of the 70 patients with gastrointestinal malignancies, esophageal, pancreatic, and hepatic flexure cancers were each present at a proportion of 5.71% (4 patients) of the cohort; rectal and sigmoid colon cancer each represented 21.43% (15 patients) of the cohort; 14.29% (10 patients) had stomach cancer; and 11.43% (8 patients) had colon cancer. In 8.57% (6 patients) of the cases, the initial tumor was in the cecum; in 4.29% (3 patients), it was in the rectosigmoid junction; and we had 1 patient (1.43%) with duodenal cancer. Most patients (75.71%, or 53 patients) were diagnosed with stage IV cancer; 14.29% of patients, or 10 patients, were in stage III; and only 7, or 10.00%, were in stage II. A substantial portion of patients (17.14%, 12 patients) had highly undifferentiated tumors (G3), the majority (70%, 49 patients) had cancers graded G2 (moderately differentiated), and only 12.86%, or 9 patients, had low-grade tumors (G1).

The patient cohort was divided into two subgroups based on the type of administered therapy. The first group included 30 patients treated with the FOLFOX chemotherapy regimen. The second group consisted of 40 patients who received the FOLFIRI regimen. The data of the demographic and nosological analysis performed on the recruited patients from both groups are presented in Table 1. According to the statistical analysis of the two patient groups presented in Table 1, there were no substantial differences in factors such as gender, age at diagnosis, age distribution, location of the primary tumor, and ECOG performance score. The only notable differences were in grading and disease staging.

Table 2 and Table 3 summarize the information concerning the peripheral blood routine and liver and kidney function for both groups of patients treated with FOLFOX and FOLFIRI. The data represent the values from the evaluation of patients’ baseline condition (before ChT) and the assessment following the first infusion of the patient’s suitability to continue with the next infusion in the treatment protocol (after ChT).

The FOLFOX regimen was associated with alterations in some of the key parameters of the study, i.e., a decrease in the white blood cell count of nearly 11% (r = 0.51; *p* = 0.0049), a 17% reduction in granulocyte count (r = 0.54; *p* = 0.0030), and an almost 15% decrease in platelet levels (r = 0.47; *p* = 0.0098). Despite some minor alterations in liver and kidney function parameters, no statistically significant changes were observed, and they remained stable.

Subjects undergoing FOLFIRI-based therapy had hemoglobin levels below the established lower threshold (116 vs. 120). Hemoglobin levels showed no statistically significant variation before or after chemotherapy when comparing FOLFOX- and FOLFIRI-treated patients. In individuals receiving treatment with the FOLFIRI regimen, again, we noted a statistically significant reduction in white blood cell count and granulocyte count, at 37% (r = 0.39; *p* = 0.0129) and 41% (r = 0.43; *p* = 0.0063), respectively. In contrast, platelet levels showed only a minor decrease of less than 2%, which was not statistically significant. The therapy did not significantly affect kidney and liver function.

Based on data presented in Table 2 and Table 3, PLR and LMR were calculated as markers of systemic inflammation (Table 4). The performed analysis denoted a lack of statistically significant differences in systemic inflammatory markers between the FOLFOX and FOLFIRI groups at either the pre- or post-infusion time points.

**Figure 1:**
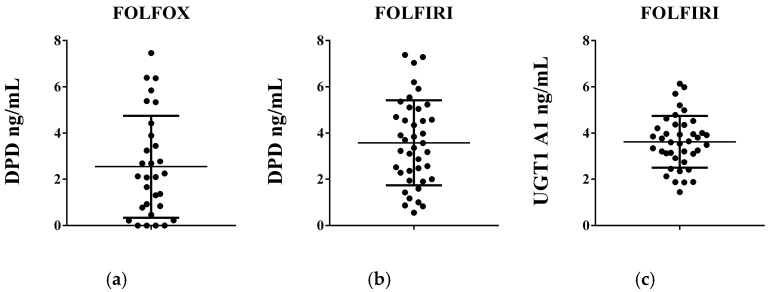
(**a**,**b**) Dihydropyrimidine dehydrogenase (DPD) and (**c**) UDP-glucuronosyltransferase (UGT1A1) enzyme levels in patients receiving the FOLFOX or FOLFIRI chemotherapeutic regimen determined by means of enzyme-linked immunosorbent assay. Plasma level is expressed as ng/mL and presented as scatter plots with the mean value and SD.

The results from the enzyme-linked immunosorbent assays denote that the enzyme levels and distribution in the studied population were found to be dependent on the type of chemotherapy regimen administered (Figure 1). Among individuals receiving treatment with FOLFOX, four patients had no detectable level of the DPD enzyme. DPD levels ranged from 0.00 ng/mL to 7.467 ng/mL, with a mean of 2.543 ng/mL and a median of 2.108 ng/mL. In the group of individuals treated with FOLFIRI, we did not observe patients with undetectable DPD or UGT1 A1 levels. The DPD enzyme level ranged from 0.555 ng/mL to 7.383 ng/mL, with a mean of 3.579 ng/mL and a median of 3.468 ng/mL. For the UGT1 A1 level, the obtained values were between 1.444 ng/mL and 6.138 ng/mL, with a mean of 3.623 ng/mL and a median of 3.628 ng/mL.

A multiple linear regression analysis was conducted to assess the possibility of the disturbance of the proper relationship between enzyme levels and toxicity outcome. Specifically, the potential influence of age, gender, and disease stage on DPD and UGT1A1 levels was assessed (Table 5). The regression model revealed no statistically significant association between the mentioned variables and enzyme levels (*p* > 0.05). However, for the DPD enzyme, disease stage approached significance (*p* = 0.086), demonstrating a borderline suggestive pattern. An additional analysis, including the progression factor following prior therapy, was also considered in the new regression model. This time, DPD levels were significantly associated with cancer stage (*p* = 0.034).

**Figure 2:**
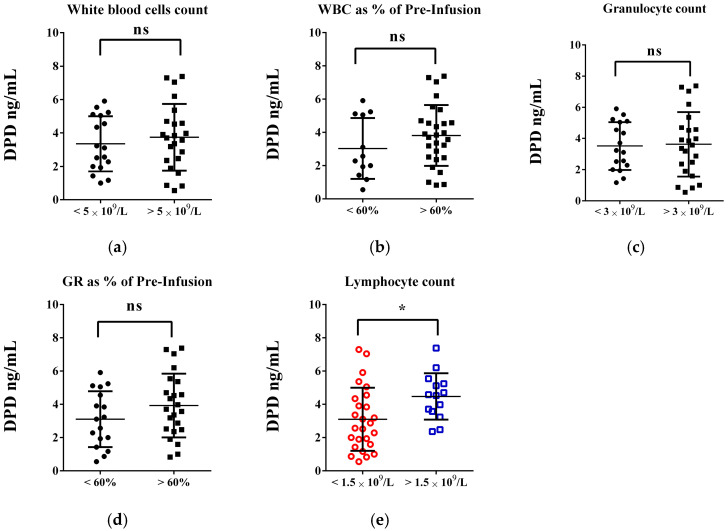
Exploring the link between DPD enzyme levels [ng/mL] and changes in basic peripheral blood routine parameters in patients on FOLFIRI chemotherapy: myelotoxicity considerations. (**a**,**b**) Statistical differences and trends related to white blood cell count and its changes. (**c**,**d**) Variations in granulocyte count and its changes. (**e**) Significant changes in lymphocyte count. An unpaired *t*-test was used to evaluate the differences in means between the two independent groups, considering *p* < 0.05 as the threshold for statistical significance (ns—not significant; * *p* < 0.05—colored graphs).

As the next step, using bivariate analysis and multiple linear regression, we explored the potential relations between the obtained level of the enzymes responsible for the metabolism of 5-FU and irinotecan and the specific baseline for peripheral blood parameters cited as potential indicators of the myelotoxic effects of FOLFIRI regimens and determined according to our previous investigations [30,31]. The data concerning DPD levels were presented in Figure 2 and Figure S1 (see in Supplementary Materials). No clear trend or consistent relationship was observed between the DPD levels of patients with a white blood cell count below 5 × 10^9^/L and a granulocyte count of 3 × 10^9^/L and the other patients on the FOLFIRI regimen—DPD values displayed a percentage reduction of around 10 and 3%, respectively (Figure 2a,c). When comparing DPD levels in patients who experienced more than a 60% reduction in these two parameters, we observed an apparent decrease in DPD values correlating with more apparent reduction effects (Figure 2b,d). On average, DPD levels decreased by approximately 20%. In this group, however, a statistically significant correlation between lymphocyte count and DPD was observed (Figure 2e). Patients with lymphocyte levels below 1.5 × 10^9^/L had significantly lower DPD levels than others (3.097 vs. 4.474 ng/mL; *p* = 0.0223; d = 0.808). No notable changes in platelet count were linked to lower DPD values.

**Figure 3:**
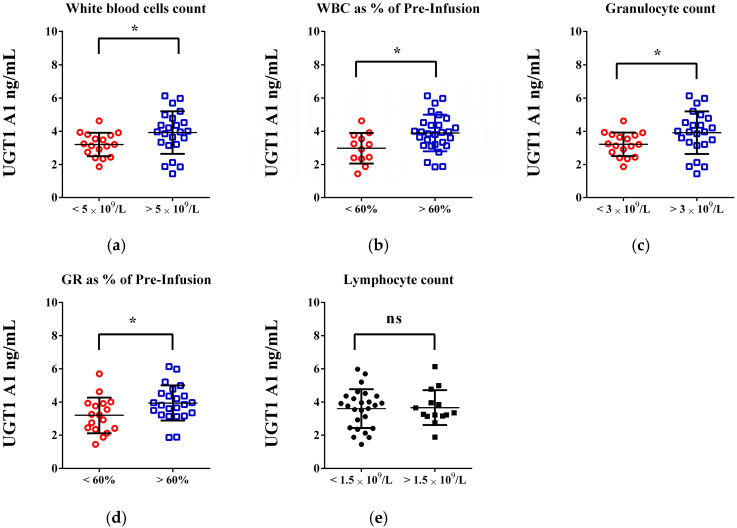
Exploring the link between UDP-glucuronosyltransferase (UGT1A1) enzyme levels [ng/mL] and changes in basic peripheral blood routine parameters in patients on FOLFIRI chemotherapy: myelotoxicity considerations. (**a**,**b**) Statistical differences and trends related to white blood cell count and its changes; (**c**,**d**) Significant differences in granulocyte count and its changes. (**e**) Changes in lymphocyte count. An unpaired *t*-test was used to evaluate the differences in means between the groups. *p* < 0.05 was considered a threshold for statistical significance (ns—not significant; * *p* < 0.05—colored graphs).

**Figure 4:**
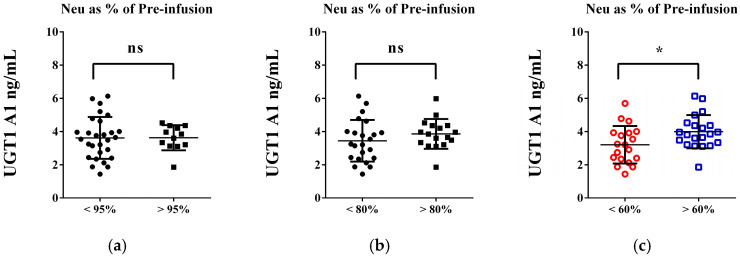
Exploring the association between UDP-glucuronosyltransferase (UGT1A1) enzyme levels [ng/mL] and changes in neutrophil count. Neu count has decreased to (**a**) 95%, (**b**) 80% and (**c**) 60% of Pre-infusion base line level. An unpaired *t*-test was used to evaluate the differences in means between the groups. *p* < 0.05 was considered a threshold for statistical significance (ns—not significant; * *p* < 0.05—colored graphs).

The changes in white blood cell (WBC) and granulocyte counts, along with their reductions from baseline levels, are associated with UGT1A1 enzyme levels in individuals receiving the FOLFIRI protocol (Figure 3). Statistically significant differences in UGT1A1 levels were observed in patients with a WBC count below 5 × 10^9^/L (3.209 vs. 3.929 ng/mL; *p* = 0.0295; d = 0.668) and a granulocyte count below 1.5 × 10^9^/L (3.218 vs. 3.922 ng/mL; *p* = 0.0487; d = 0.65) compared to those with higher values (Figure 3a,c). In patients experiencing a 60% or more significant reduction in these counts, UGT1A1 levels decreased by approximately 20%. No notable changes in lymphocyte count or platelets were linked to lower UGT1 A1 values (Figure 3e). The relationship between UGT1A1 levels and the decrease in neutrophils, expressed as a percentage of their baseline value, is shown in Figure 4.

The results from the multiple linear regression analysis evaluating the associations between DPD and UGT1A1 levels and hematological outcomes after chemotherapy are presented in Table 6 and Table S1 (see in Supplementary Materials). In the three models concerning enzyme levels, with white blood cell, granulocyte, and neutrophil counts determined in the second blood sample as dependent variables, the *p*-values approach statistical significance. For WBC as a dependent variable, a lack of statistical significance was observed (F(2, 37) = 2.42, *p* = 0.103). It explained approximately 11.6% of the variance in WBC (R^2^ = 0.116) and only UGT1A1 showed a significant positive effect (b* = 0.359, *p* = 0.042). We observed the model’s overall fit, investigating granulocytes and neutrophils as dependent variables. Both of them were marginally not significant (F(2, 37) = 2.71, *p* = 0.081), explaining 12.8% of the variance (R^2^ = 0.128). Again, the predictor, UGT1A1, emerged with a statistically significant positive effect (b* = 0.383, *p* = 0.030). The lymphocyte model and models associated with the estimation of the effect of both predictors on data presented as a percentage of reinfusion had minimal exploratory power (R^2^ < 0.020) and lacked statistical significance.

## 4. Discussion

The current work presents results from a retrospective single-center study with fixed sample sizes. This has consequently led to relatively limited and moderate patient group sizes (70, with 30 in the FOLFOX group and 40 in the FOLFIRI group). The performed statistical analysis of both groups of patients presented in Table 1 revealed no significant differences across the various indicators such as gender, age at diagnosis, age-related distribution, primary tumor location, and ECOG performance status. The only significant differences were in grading and disease staging. In the second group, where patients were treated with FOLFIRI, all patients were in stage IV, while in the FOLFOX group, there were patients in stages II and III of the disease. This result is not due to poor patient selection but to the specificity and recommendations regarding applying the two chemotherapy regimens depending on the patient’s general condition, the expected side effects, and the grading of the initial tumor. Even though both regimens have often been described as comparable in terms of their progression-free survival benefits for the treatment of advanced colorectal cancer, FOLFOX has been cited in the scientific literature as being more popular, especially after 2007, and with time, the oxaliplatin-based regimen became the standard of first-line care for stage III and IV cancer [32]. FOLFIRI is widely accepted for use as the standard second-line option when oxaliplatin treatment is no longer effective [33]. Moreover, both groups are representative samples that reflect the global trends in the development and distribution of gastrointestinal tumors. The distribution of male and female patients in our study was nearly equal, consistent with the tendency for a slightly elevated incidence of gastrointestinal malignancies in men compared to women. In our study, the patient age distribution mirrored the expected trend of greater disease susceptibility in older individuals—more than 65% of patients were aged between 60 and 80. Additionally, a concerning rise in younger patients was evident, with more than 10% being under the age of 50, in line with published data [34].

On day fourteen of the chemotherapeutic infusion, both groups of patients exhibited characteristic side effects, especially those associated with myelotoxicity. This is in accordance with the established clinical practice concerning the nadir period (7–12 days post-infusion, lasting 5–7 days) [35,36]. For FOLFOX patients, the characteristic 5-FU-induced statistically significant decreases in the white blood cell count, granulocyte count, and platelets were observed. In patients who are administered the FOLFIRI regimen, the combination of 5-fluorouracil (5-FU) with other antineoplastic agents with similar adverse effect profiles, such as irinotecan, is expected to modulate the specific myelosuppressive effects [37]. In our study, the cumulative impact on hematologic toxicity led to a decrease in white blood cell count, granulocytes, neutrophils, and monocytes. This indicates that the two chemotherapy regimens significantly affect the hematological profiles, though their degree of impact varies. The cumulative impact of 5-FU and irinotecan results in a greater absolute reduction in WBCs and granulocytes of more than one-third. Although the *p*-values were below 0.05, indicating statistical significance, the results for effect size revealed moderate and more consistent impacts in FOLFOX compared to FOLFIRI. Despite the discussed high magnitude of reduction, this observation was associated with a smaller effect size. This likely reflects the variability in patients’ response, i.e., elevated post-treatment standard deviations in the FOLFIRI group, which comprises metastatic cancer patients who experienced progression following prior treatment. Although *p*-values exceeded 0.05 in the bivariate analysis concerning the possibility of interference between systemic inflammatory factors and hematologic parameters due to the increased tumor burden and immune dysregulation in stage IV cancer, some trends have been observed. These non-significant changes suggest a more active systemic inflammation state, consistent with stage IV disease. Notably, while PLR decreased following chemotherapy in the FOLFOX patient group, it increases in the FOLFIRI group. Additionally, both the FOLFOX and FOLFIRI groups exhibited a post-chemotherapy upward trend in LMR, but the values in the FOLFIRI group remained consistently lower.

Identifying factors associated with responsiveness, resistance, and a predisposition of patients to develop severe adverse reactions has emerged as an increasingly important area of research. Numerous approaches have been investigated in an effort to accurately assess enzyme deficiency or dysfunction before treatment to avoid early and severe toxicity. These include genetic screening for polymorphisms, phenotypic assays to determine enzyme activity, measuring enzyme quantity through ELISA assays, and mathematical models [38,39,40]. Many challenges and restrictions are associated with their use. Genetic testing reveals known polymorphisms but it does not take into account post-translational modifications and expression regulation. Phenotypic screening is a more accurate representation of enzyme activity but has no standardized and regulatory-approved methods and relies only on guidelines and recommendations. The main limitation of the ELISA method used in this study when determining DPD and UGT1A1 levels is that it only quantifies proteins [41]. The possibility of a discrepancy between protein levels and actual enzymatic activity exists, resulting from epigenetic regulation and enzyme inhibition.

Our results concerning the DPD level in patients assigned for FOLFOX were in accordance with data from other authors who have used alternative methods to study this parameter [42]. The established DPD levels varied within a wide concentration range, and we observed the division of individuals into separate groups. DPD levels of patients treated with FOLFIRI chemotherapy also displayed a wide range of values, but no data clustering or formation of potential subgroups was observed. Comparing the DPD levels of both groups of patients assessed for different chemotherapeutic regimens, it is evident that patients on FOLFOX have significantly lower enzyme levels than patients on FOLFIRI (2.543 vs. 3.579, *p* = 0.0363). In the group receiving the FOLFIRI regimen, 97.5% of the patients were previously on another chemotherapeutic treatment, and only one patient received it as a first line of therapy due to diabetic polyneuropathy. Due to disease progression observed in the remaining 39 patients, the treatment course was changed to FOLFIRI.

The DPD regression model, including three predictors—age, cancer stage and gender—with 70 observations was not statistically significant (F(3, 66) = 1.492, *p* = 0.223). This suggests that, taken together, the predictors do not significantly explain variations in the dependent variable (DPD). Among the predictors, the cancer stage has a borderline *p*-value of 0.086. Given the limited number of patients and the small to moderate effect size (b* = 0.211), we refined the model. We did not only consider the stage of the disease but also its progression after earlier therapy administration. Stage IV cancer patients were divided into two groups: the first group included those receiving initial treatment and the second group included patients whose disease had progressed after previous chemotherapy. This improvement in variable definition enhanced the model’s sensitivity, identifying disease progression as a significant individual predictor. The simplified regression model, comprising only disease progression, indicated that clinical stage is a statistically significant predictor of DPD when considered independently with a small to moderate positive relationship (b* = 0.258).

DPD deficiency has been related to severe toxic effects after the administration of 5-FU [42,43]. On the other hand, high DPD activity has been cited as resulting in low 5-FU circulating levels, which could subsequently induce a relative reduction in treatment efficacy [44,45]. A much wider range of DPD gene expression in patients who did not benefit from treatment compared to those who showed favorable outcomes with intravenous 5-FU/leucovorin for advanced colorectal cancer has been observed [46]. Increased levels of the DPD enzyme after oxaliplatin administration associated with treatment resistance have also been reported [47]. DPD enzyme expression and its genetic regulation have been explored in depth. The reported findings by different scientific groups over the years have provided contradictory results regarding the use of enzyme levels as a biomarker for efficacious response to the applied treatment. Some authors related lower DPD levels to poorer patient survival and presented data proving an inverse relationship between the level of the enzyme and the proliferative activity and expression of p53 in tumor cells [48,49].

The results obtained from the bivariate analysis and multivariate regression show that UGT1A1 is the most potent factor in the determination of treatment-related changes in the hematological parameters. In the bivariate analysis, significant differences (*p* < 0.05) have been observed when comparing its level between groups displaying different absolute values of the studied parameters and also in cases where they are presented as relative values (percentage of each patient’s baseline). The multivariate regression, on the other hand, displays associations only with the absolute count of the studied parameters (which is usually used by clinicians in medical practice). UGT1A1 emerged as a moderate positive predictor, with standardized beta coefficients of approximately 0.38 and *p*-values around 0.030–0.042. The used models had modest explanatory power. Their R^2^ values ranged from 0.116 to 0.128, indicating that the used markers accounted for just 11–13% of the variability.

In our study, we investigated the relationship between the observed levels of the studied enzymes and the changes in some peripheral blood parameters. In the FOLFIRI group, where the myelotoxic effects of low DPD and low UGT1A1 expression are expected to partially overlap, suggesting that combined deficiencies may exacerbate hematologic toxicity in a synergistic manner, the changes in the studied parameters seem to correlate more strongly with UGT1A1 levels, with no noticeable association with DPD (the high DPD expression likely reduces its influence on toxicity outcomes, thereby explaining the lack of a strong association). These observations should be cautiously interpreted within the limitations of a retrospective, single-center design, a relatively small sample size, and population heterogeneity. Despite the consistent pattern reaching statistical significance identified during data analysis, this study’s statistical power might be insufficient to detect smaller effects reliably, and there is a risk of type II errors. Further investigations in larger, prospective studies are needed to validate the observed associations and help to determine their potential.

## 5. Conclusions

In conclusion, our study demonstrated that patients on both regimens presented with the typical alterations associated with their application—reductions in white blood cell count, granulocyte count, and platelets for FOLFOX and decreases in white blood cell count, granulocyte count, and neutrophils in FOLFIRI. A lack of post-infusional statistical significance in red blood cells, hemoglobin, and hematocrit was observed. Liver and kidney function remained stable during treatment. The association of DPD levels with cancer progression was observed. In the FOLFIRI group, the changes seem to correlate with UGT1A1 levels. Further investigation and genotyping in the group of patients with low DPD and UGT1A levels is warranted to better understand the correlations between the phenotypes and genotypes related to chemotherapy-related adverse events.

## Figures and Tables

**Table 1:** Descriptive profile of demographic, clinical, and pathological characteristics of both studied groups.

	FOLFOX *n* = 30, (%)	FOLFIRI *n* = 40, (%)	*p*-Values
**Gender**			**ns**
Male	**16** (53.3%)	**22** (55.0%)	
Female	**14** (46.7%)	**18** (45.0%)	
**Age at diagnosis**			**ns**
Average	63 y, 9 mo.	63 y, 6 mo.	
Range	43 ↔ 80	34 ↔ 83	
**Age-Related Distribution**			**ns**
Below 50 years	**4** (13.3%)	**5** (12.5%)	
Between 50 and 59	**6** (20.0%)	**5** (12.5%)	
Between 60 and 69	**8** (26.7%)	**13** (32.5%)	
Between 70 and 79	**11** (36.7%)	**14** (35.0%)	
Above 80 years	**1** (3.3%)	**3** (7.5%)	
**Primary Tumor Location**			**ns**
Ca. oesophagi	**2** (6.7%)	**2** (5.0%)	
Ca. ventriculi	**1** (3.3%)	**9** (22.5%)	
Ca. glandulae pancreaticae	**0** (0.0%)	**4** (10.0%)	
Ca. recti	**9** (30.0%)	**6** (15.0%)	
Ca. rectosigmoidei	**1** (3.3%)	**2** (5.0%)	
Ca. sigmoidei	**9** (30.0%)	**6** (15.0%)	
Ca. flexurae hepatis	**0** (0.0%)	**4** (10.0%)	
Ca. coli	**5** (16.7%)	**3** (7.5%)	
Ca. cecum	**3** (10.0%)	**3** (7.5%)	
Ca. duodeni	**0** (0.0%)	**1** (2.5%)	
**Grading**			**
Well differentiated (G1)	**7** (23.3%)	**2** (5.0%)	
Moderately differentiated (G2)	**22** (73.3%)	**27** (67.5%)	
Poor/undifferentiated (G3)	**1** (3.3%)	**11** (27.5%)	
**Disease Stage**			****
Stage I	**0** (0%)	**0** (0%)	
Stage II	**7** (23.3%)	**0** (0%)	
Stage III	**10** (33.3%)	**0** (0%)	
Stage IV	**13** (43.3%)	**40** (100.0%)	
**ECOG performance status**			**ns**
0	**2** (6.7%)	**0** (0.0%)	
1	**20** (66.7%)	**26** (65.0%)	
2	**8** (26.6%)	**13** (32.5%)	
3	**0** (0%)	**1** (2.5%)	

Notes: The χ^2^ test was used for categorical data analysis, and results were deemed statistically significant when *p* < 0.05 (ns—not significant; ** *p* < 0.01; **** *p* < 0.0001).

**Table 2:** Pre- and post-treatment biochemical and hematological profiles in FOLFOX chemotherapy (ChT).

Parameter		Before ChT (Median; IQR)	After ChT (Median; IQR)	*p*-Value (Wilcoxon Test)
**Peripheral blood routine**				
WBC	10^9^/L	**7.15; _(5.50; 8.40)_**	**6.35; _(4.23; 7.60)_**	**
LY	10^9^/L	1.64; _(1.36; 1.99)_	1.66; _(1.25; 1.99)_	ns
MO	10^9^/L	0.48; _(0.36; 0.63)_	0.41; _(0.33; 0.60)_	ns
GR	10^9^/L	**5.30; _(3.52; 6.09)_**	**4.39; _(2.30; 4.94)_**	**
RBC	10^12^/L	4.35; _(3.83; 4.59)_	4.26; _(3.97; 4.69)_	ns
HGB	g/dL	121.0; _(111.5; 130.3)_	124.0; _(108.8; 135.5)_	ns
HCT	L/L	37.40; _(33.68; 39.25)_	37.10; _(33.63; 40.00)_	ns
PLT	10^9^/L	**280.0; _(243.5; 347.5)_**	**244.0; _(183.5; 324.5)_**	**
**Liver functions**				
ALAT	U/L	19.70; _(13.20; 31.63)_	16.80; _(11.45; 25.33)_	ns
ASAT	U/L	20.05; _(17.53; 31.48)_	19.20; _(15.93; 25.15)_	ns
TBIL	µmol/L	8.95; _(7.00; 15.18)_	10.80; _(7.83; 15.00)_	ns
**Renal functions**				
Cr	µmol/L	81.50; _(69.75; 89.25)_	80.80; _(72.00; 92.50)_	ns

Note: Results were expressed as the median and interquartile range (IQR). The Wilcoxon signed-rank test was employed to evaluate paired differences between pre- and post-FOLFOX chemotherapy values, with statistical significance set at *p* < 0.05 (ns—not significant; ** *p* < 0.01); WBC—white blood cells; LY—lymphocytes, absolute; MO—monocytes, absolute; GR—granulocytes, absolute; RBC—red blood cells; HGB—hemoglobin; HCT—hematocrit; PLT—platelets; ALAT—alanine aminotransferase; ASAT—aspartate aminotransferase; TBIL—total bilirubin; Cr—creatinine.

**Table 3:** Pre- and post-treatment biochemical and hematological profiles in FOLFIRI chemotherapy (ChT).

Parameter		Before ChT (Median; IQR)	After ChT (Median; IQR)	*p*-Value (Wilcoxon Test)
**Peripheral blood routine**				
WBC	10^9^/L	**8.30; _(5.69; 9.82)_**	**5.24; _(3.74; 9.51)_**	*
LY	10^9^/L	1.39; _(1.07; 1.86)_	1.28; _(0.98; 1.68)_	ns
MO	10^9^/L	**0.58; _(0.42; 0.76)_**	**0.43; _(0.33; 0.66)_**	*
GR	10^9^/L	**6.00; _(3.74; 7.83)_**	**3.51; _(2.01; 7.21)_**	**
RBC	10^12^/L	4.00; _(3.60; 4.39)_	3.96; _(3.56; 4.31)_	ns
HGB	g/dL	116.0; _(105.0; 129.8)_	116.5; _(102.0; 128.0)_	ns
HCT	L/L	35.25; _(32.23; 39.00)_	36.10; _(32.03; 38.23)_	ns
PLT	10^9^/L	243.5; _(178.0; 350.8)_	238.5; _(160.5; 346.5)_	ns
**Liver functions**				
ALAT	U/L	14.40; _(11.13; 25.40)_	15.24; _(11.25; 28.40)_	ns
ASAT	U/L	24.80; _(18.75; 35.53)_	27.05; _(18.05; 33.68)_	ns
TBIL	µmol/L	10.05; _(7.50; 17.55)_	10.20; _(7.18; 17.90)_	ns
**Renal functions**				
Cr	µmol/L	80.00; _(73.00; 90.75)_	75.00; _(69.25; 93.25)_	ns

Note: Results were expressed as the median and interquartile range. The Wilcoxon signed-rank test was employed to evaluate paired differences between pre- and post-FOLFIRI chemotherapy values, with statistical significance set at *p* < 0.05 (ns—not significant; * *p* < 0.05; ** *p* < 0.01); WBC—white blood cells; LY—lymphocytes, absolute; MO—monocytes, absolute; GR—granulocytes, absolute; RBC—red blood cells; HGB—hemoglobin; HCT—hematocrit; PLT—platelets; ALAT—alanine aminotransferase; ASAT—aspartate aminotransferase; TBIL—total bilirubin; Cr—creatinine.

**Table 4:** Pre- and post-treatment platelet-to-lymphocyte ratio (PLR) and lymphocyte-to-monocyte ratio (LMR) in patients on FOLFOX and FOLFIRI.

Parameter	FOLFOX (Median; IQR)	FOLFIRY (Median; IQR)	*p*-Value (Mann–Whitney U Test)
**Platelet-to-lymphocyte ratio**			
PLR Before ChT	181.0; _(135.7; 210.4)_	168.7; _(123.6; 256.4)_	ns
PLR After ChT	153.7; _(108.6; 223.1)_	186.4; _(120.5; 270.4)_	ns
**Lymphocyte-to-monocyte ratio**			
LMR Before ChT	3.643; _(2.772; 4.265)_	2.548; _(2.070; 3.759)_	ns
LMR After ChT	3.738; _(3.106; 5.190)_	2.998; _(2.328; 4.607)_	ns

**Table 5:** Multiple linear regression analysis on DPD and UGT1A1 enzyme levels in relation to demographic characteristics, cancer stage, and progression.

Parameter	b*	Std. Error of b*	b	Std. Error of b	t	*p*-Value
Regression Summary for Dependent Variables: DPD (age, gender, disease stage);						
*R* = 0.252; *R*^2^ = 0.064; Adjusted *R*^2^ = 0.021						
F(3, 66) = 1.492; *p* < 0.225; STD. Error of estimate: 2.037						
*N* = 70; t(66)						
Intercept			0.428	1.907	0.224	0.823
Age	0.074	0.121	0.013	0.021	0.609	0.545
Gender	−0.0900	0.119	−0.369	0.489	−0.756	0.455
Cancer stage	0.211	0.121	0.662	0.379	0.745	0.086
Regression Summary for Dependent Variables: DPD (age, gender, disease progression);						
*R* = 0.292; *R*^2^ = 0.085; Adjusted *R*^2^ = 0.044						
F(3, 66) = 2.057; *p* < 0.114; STD. Error of estimate: 2.013						
*N* = 70; t(66)						
Intercept			0.673	1.709	0.394	0.694
Age	0.075	0.119	0.013	0.021	0.634	0.528
Gender	−0.091	0.118	−0.375	0.483	−0.776	0.441
Disease progression	0.258	0.119	0.513	0.237	2.169	**0.034**
Regression Summary for Dependent Variables: DPD (disease progression);						
*R* = 0.262; *R*^2^ = 0.069; Adjusted *R*^2^ = 0.055						
F(1, 68) = 5.029; *p* < 0.282; STD. Error of estimate: 2.001						
*N* = 70; t(68)						
Intercept	-	-	0.935	1.010	0.925	0.358
Disease stage	0.262	0.117	0.520	0.232	2.243	**0.028**
Regression Summary for Dependent Variables: UGT1A1 (age, gender)						
*R* = 0.328; *R*^2^ = 0.107; Adjusted *R*^2^ = 0.059						
F(2, 37) = 2.224; *p* < 0.1222; Std. Error of estimate: 1.089						
*N* = 40; t(36)						
Intercept			2.257	1.1170	2.021	0.051
Age	0.293	0.156	0.027	0.014	1.879	0.068
Gender	−0.126	0.156	−0.280	0.347	−0.806	0.426

Note: b*—standardized regression coefficient; Std. error of b*—standard error of the standardized regression coefficient; b—unstandardized regression coefficient; Std. error of b—standard error of the unstandardized regression coefficient; t(66)—t-statistics with 66 degrees of freedom; t(36)—t-statistics with 36 degrees of freedom. Due to the fact that all patients on FOLFIRI are in stage IV, this parameter was not included in the model.

**Table 6:** Multiple linear regression analysis of some basic hematological parameters in relation to DPD and UGT1A1 enzyme levels.

Parameter	b*	Std. Error of b*	b	Std. Error of b	t	*p*-Value
Regression Summary for Dependent Variables: WBC After (DPD, UGT1A1);						
*R* = 0.340; *R*^2^ = 0.116; Adjusted *R*^2^ = 0.068						
F(2, 37) = 2.421; *p* < 0.103; STD. Error of estimate: 3.743						
*N* = 40; t(37)						
Intercept			2.386	2.058	1.159	0.254
DPD	−0.053	0.171	−0.111	0.359	−0.308	0.760
UGT1A1	0.359	0.171	1.240	0.589	2.105	**0.042**
Regression Summary for Dependent Variables: Gran after (DPD, UGT1A1);						
*R* = 0.357; *R*^2^ = 0.128; Adjusted *R*^2^ = 0.081						
F(2, 37) = 2.708; *p* < 0.0800; STD. Error of estimate: 3.293						
*N* = 40; t(37)						
Intercept			0.779	1.811	0.430	0.669
DPD	−0.076	0.169	−0.141	0.316	−0.446	0.658
UGT1A1	0.383	0.169	1.171	0.518	2.259	**0.030**
Regression Summary for Dependent Variables: Neu count after (DPD, UGT1A1);						
*R* = 0.357; *R*^2^ = 0.128; Adjusted *R*^2^ = 0.081						
F(2, 37) = 0.271; *p* < 0.080; STD. Error of estimate: 3.287						
*N* = 40; t(37)						
Intercept			0.656	1.807	0.363	0.719
DPD	−0.076	0.169	−0.142	0.315	−0.449	0.656
UGT1A1	0.383	0.169	1.169	0.517	2.260	**0.030**
Ly count after (DPD, UGT1A1)***;***						
*R* = 0.089; *R*^2^ = 0.008; Adjusted *R*^2^ = _0.005						
F(2, 37) = 0.149; *p* < 0.862; STD. Error of estimate: 0.975						
*N* = 40; t(37)						
Intercept			1.416	0.536	2.642	0.012
DPD	0.098	0.181	0.051	0.093	0.544	0.590
UGT1A1	−0.035	0.181	−0.030	0.153	−0.194	0.847

Note: b*—standardized regression coefficient; Std. error of b*—standard error of the standardized regression coefficient; b—unstandardized regression coefficient; Std. error of b—standard error of the unstandardized regression coefficient; t(37)—t-statistics with 37 degrees of freedom.

## Data Availability

Additional data supporting these findings are available from the corresponding author upon reasonable request. Data sharing will be conducted in accordance with applicable privacy regulations and appropriate safeguards. For more information or specific inquiries, please contact the corresponding author.

## Supplementary Materials

The following supporting information can be downloaded at https://www.mdpi.com/article/10.3390/life15071071/s1, Figure S1: Exploring the link between dihydropyrimidine dehydrogenase (DPD) enzyme levels [ng/mL] and peripheral blood routine changes in FOLFOX chemotherapy, Table S1: Multiple linear regression analysis of some basic hematological parameters in relation to DPD and UGT1A1 enzymes levels.

## Abbreviations

ALAT	Alanine aminotransferase
ASAT	Aspartate aminotransferase
DPD	Dihydropyrimidine dehydrogenase
FOLFOX	Folinic acid, fluorouracil and oxaliplatin
FOLFIRI	Folinic acid, fluorouracil and irinotecan
GR	Granulocytes, absolute
ChT	Chemotherapy
Cr	Creatinine
HCT	Hematocrit
HGB	Hemoglobin
LY	Lymphocytes, absolute
MO	Monocytes, absolute
PLT	Platelets
RBC	Red blood cells
TBIL	Total bilirubin
UGT1A1	UDP-glucuronosyltransferase
WBC	White blood cells
5-FU	Fluorouracil
