# Supplementary material for: Gastrointestinal Cancers with Consideration of DPD and UGT1A1 Plasma Levels: Chemotherapy-Related Toxicity
Source: Life (Basel). 2025 Jul 4;15(7):1071. doi: 10.3390/life15071071 (PMC12300698; doi:10.3390/life15071071)
Supplement: Supplementary file 1 [file life-15-01071-s001.zip › life-3654188-supplementary.pdf]

life

MDPI

Article

Gastrointestinal Cancers with Consideration of DPD and UGT1A1 Plasma Levels: Chemotherapy-Related Toxicity

(Supplementary Materials)

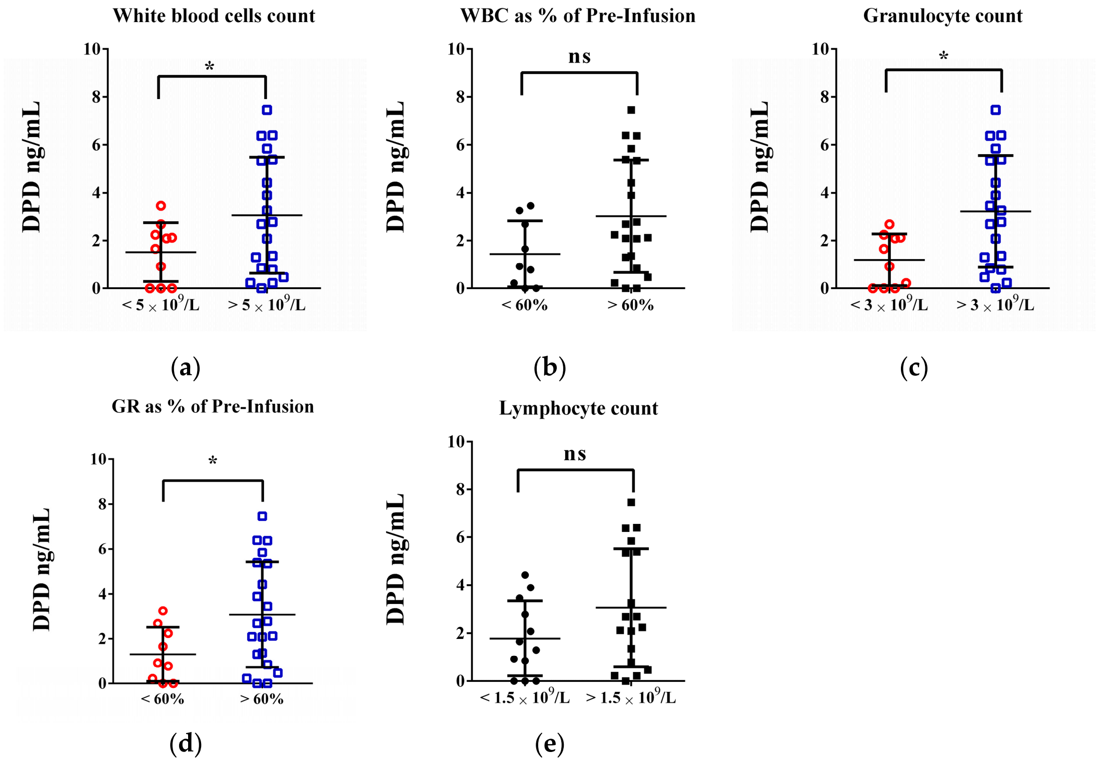

Figure S1. Exploring the link between dihydropyrimidine dehydrogenase (DPD) enzyme levels [ng/mL] and peripheral blood routine changes in FOLFOX chemotherapy: myelotoxicity considerations. An unpaired t-test was applied to compare the means between the two independent groups, with statistical significance set at $p < 0.05$ (ns—not significant; * $p < 0.05$ – colored graphs). (a,b) Statistical differences and trends related to white blood cells count and its changes. Patients having a WBC below 5×10^9/L had significantly lower mean DPD levels compared to the other ($p = 0.0289$; d = 0.727). (c,d) A significant decrease in DPD levels observed in patients with a granulocyte count below 3×10^9/L ($p = 0.0152$; d = 1.002) and those with a 60% or more significant reduction in their granulocyte count ($p = 0.0423$; d = 0.847). (e) A non-significant increase in DPD levels near the lower limit in lymphocyte count of 1.5×10^9/L.

Table S1: Multiple linear regression analysis of some basic hematological parameters in relation to DPD and UGT1A1 enzymes levels.

**Parameter**	**b***	**Std. error of b***	**b**	**Std. error of b**	**t**	**p-value**
Regression Summary for Dependent Variables: WBC % of preinfusion (DPD, UGT1A1); $R = 0.132$; $R^2 = 0.017$; Adjusted $R^2 = 0.009$ F(2.37)=0.329; $p < 0.722$; STD. Error of estimate: 86.415 $N = 40$; t(37)						
Intercept			67.767	47.515	1.426	0.162
DPD	-0.076	0.1800	-3.475	8.282	-0.4200	0.677
UGT1A1	0.145	0.1800	10.961	13.601	0.806	0.426
Regression Summary for Dependent Variables: Gran % of reinfusion (DPD, UGT1A1); $R = 0.104$; $R^2 = 0.0108$; Adjusted $R^2 = 0.006$ F(2.37) = 0.202; $p < 0.818$; STD. Error of estimate: 147.86 $N = 40$; t(37)						
Intercept			66.530	81.302	0.818	0.418
DPD	-0.060	0.180	-4.722	-14.171	-0.333	0.741
UGT1A1	0.114	0.180	14.696	23.273	0.631	0.532
Regression Summary for Dependent Variables: Neu % of preinfusion (DPD, UGT1A1) $R = 0.106$; $R^2 = 0.011$; Adjusted $R^2 = 0.006$ F(2.37) = 0.212; $p < 0.810$; STD. Error of estimate: 159.41 $N = 40$; t(37)						
Intercept	-	-	67.398	87.652	0.769	0.447
DPD	-0.068	0.180	-5.679	15.278	-0.372	0.712
UGT1A1	0.116	0.180	16.099	25.091	0.642	0.525

Note: b*—standardized regression coefficient; Std. error of b*—standard error of the standardized regression coefficient; b—unstandardized regression coefficient; Std. error of b—standard error of the unstandardized regression coefficient; t(37)—t-statistics with 37 degrees of freedom.
